# Adolescent fertility and family planning in East Asia and the Pacific: a review of DHS reports

**DOI:** 10.1186/1742-4755-8-11

**Published:** 2011-05-05

**Authors:** Elissa Kennedy, Natalie Gray, Peter Azzopardi, Mick Creati

**Affiliations:** 1 Centre for International Health, Burnet Institute, Melbourne Australia; 2 Department of Epidemiology and Preventive Medicine, Monash University, Melbourne Australia; 3 Centre for Adolescent Health, Royal Children's Hospital, Melbourne Australia

## Abstract

**Background:**

Adolescent pregnancy has significant health and socio-economic consequences for women, their families and communities. Efforts to prevent too-early pregnancy rely on accurate information about adolescents' knowledge, behaviours and access to family planning, however available data are limited in some settings. Demographic and Health Survey (DHS) reports are recognised as providing nationally representative data that are accessible to policymakers and programmers. This paper reviews DHS reports for low and lower middle income countries in East Asia and the Pacific to determine what information regarding adolescent fertility and family planning is available, and summarises key findings.

**Methods:**

The most recent DHS reports were sought for the 33 low and lower middle income countries in the East Asia and Pacific region as defined by UNICEF and World Bank. Age-disaggregated data for all indicators relevant to fertility and current use, knowledge and access to family planning information and services were sought to identify accessible information. Reported data were analysed using an Excel database to determine outcomes for adolescents and compare with adult women.

**Results:**

DHS reports were available for eleven countries: Cambodia, Indonesia, Marshall Islands, Nauru, Papua New Guinea, Philippines, Samoa, Solomon Islands, Timor-Leste, Tuvalu and Vietnam. Twenty seven of 40 relevant DHS indicators reported outcomes for adolescent women aged 15-19 years. There were limited data for unmarried adolescents. A significant proportion of women commence sexual activity and childbearing during adolescence in the context of low contraceptive prevalence and high unmet need for contraception. Adolescent women have lower use of contraception, poorer knowledge of family planning and less access to information and services than adult women.

**Conclusion:**

DHS reports provide useful and accessible data, however, they are limited by the failure to report data for unmarried adolescents and report age-disaggregated data for some indicators. Further research is required to better understand the barriers that both married and unmarried adolescents face accessing reproductive health information and services, and their information and service delivery preferences.

## Background

There are over 14 million births to adolescent women aged 15-19 each year, 91 percent of these in low and middle-income countries [1]. Six million adolescent pregnancies are unintended and occur in the context of low contraceptive prevalence [2,3]. Less than one third of currently married adolescent women in low and middle income countries who want to avoid pregnancy are using a modern method of contraception, and more than 60 percent would like to avoid or delay pregnancy but are not able to do so [4]. Less is known about unmarried adolescents. Sexual activity outside of marriage is increasing, but less than half of those who want to avoid pregnancy are using a modern method of contraception [4-6].

Adolescent pregnancy carries an increased risk of adverse health outcomes for young women and their children. Globally, adolescents account for eleven percent of all births but contribute to 23 percent of the burden of disease related to pregnancy and childbirth. Adolescents aged 10-14 years are five times more likely to die as a result of pregnancy and childbirth than adult women, and maternal conditions are the leading cause of death among women aged 15-19 [7-9]. Adolescents account for around 14 percent of unsafe abortions, an estimated 2.5 million every year [2,10]. Babies of adolescent mothers have a 50-100 percent increased risk of mortality within the first month of life and suffer higher rates of perinatal morbidity compared with infants born to adult women [8,11]. By impacting on education, employment and economic opportunities, pregnancy during adolescence can also have lasting socio-economic consequences which, in turn, contribute to poorer health outcomes, gender inequity and poverty of adolescent mothers, their families and communities [12-15].

Preventing adolescent pregnancy is an essential component of a comprehensive approach to improve adolescents' sexual and reproductive health, which should also include efforts to address adolescents' vulnerability to sexually transmitted infections, HIV and gender-based violence. Developing evidence-based strategies to prevent too-early pregnancy requires policymakers and program designers to have access to data on adolescents' reproductive health, behaviours, and utilisation of family planning information and services. Much of the available literature on contraceptive use among adolescent women focuses on sub-Saharan Africa, Latin America and South Asia [3,6,16,17], while relatively less is known about access to family planning in East Asia and, in particular, the Pacific. The recent completion of Demographic and Health Surveys (DHS) in a number of countries in this region presents an opportunity to begin to fill this gap. DHS reports are recognised as providing valuable and accessible information to policymakers and programmers in low and middle income countries [18]. They are also one of the most comprehensive sources of national-level data that capture reproductive health information, including data on adolescents [19].

This paper reviews DHS reports for low and lower middle income countries in East Asia and the Pacific to identify accessible information about adolescent fertility and current use, knowledge and access to family planning information and services. It also summarises key findings from available data to provide an overview of adolescent fertility and family planning in the region, and identifies potential targets for further research to better inform policy and programs.

## Methods

The most recent DHS reports were sought for the 33 low and lower middle income countries in the East Asia and Pacific region as defined by UNICEF and World Bank geographic region and income group classifications [20,21]. DHS reports were identified from Measure DHS [22], the Secretariat of the Pacific Community (SPC) [23] and through general internet searches.

Available DHS reports were reviewed to identify all indicators relevant to: sexual activity, fertility and unintended pregnancy; contraceptive prevalence and unmet need for contraception; knowledge of family planning and exposure to family planning information; and problems accessing health services. Age-disaggregated data for each of these indicators were sought from the DHS reports to determine what information about adolescent women (aged 10-19 years) is accessible to policymakers and programmers. All reported data disaggregated by age were entered into an Excel [24] database to identify outcomes for adolescents and, where possible, compare with adult women for each country.

Data disaggregated by marital status in addition to age were also sought. However, while the surveys employ a standard methodology, the sampled population varies between countries, particularly in relation to eligibility for the "Women's Questionnaire" based on marital status. Some countries survey 'ever-married women' only, defined as women who are currently married or living in a consensual union and those who are divorced, widowed or separated. Other countries survey 'all women' including ever-married and unmarried women [22].

The review was limited to adolescent females, but it is acknowledged that many DHS also report outcomes for adolescent males for some indicators.

## Results

DHS reports were available for eleven countries: Cambodia [25], Indonesia [26], Marshall Islands [27], Nauru [28], Papua New Guinea [29], Philippines [30], Samoa [31], Solomon Islands [32], Timor-Leste [33], Tuvalu [34] and Vietnam [35]. Thailand was excluded because the survey was conducted more than 20 years ago. Sampling periods of the DHS reports ranged from 1996 to 2009.

Forty relevant indicators were identified from the DHS reports (Table 1). Twenty seven of these were age-disaggregated to report data for women aged 15-19. No data were reported for adolescents aged 10-14. The number of countries reporting data for each indicator varied due to different survey methodologies, but age-disaggregated data were available for all countries for twelve indicators.

**Table 1:** Identified indicators from DHS reports

Indicators reporting data for women aged 15-19 for all countries	Indicators reporting data for women aged 15-19 for at least one country	Indicators reporting data for both married and unmarried women aged 15-19 for at least one country	Indicators not reporting data for women aged 15-19
Current marital status Age at first marriage Age-specific fertility rate Trends in age-specific fertility rate Age first birth Children ever born and living Birth intervals Teenage childbearing Fertility planning Ever use of contraception Current use of contraception Unmet and met need for contraception	Age at first sex Recent sexual activity Timing of first birth Fertility preferences Number of children at first use of contraception Quality of use/compliance with contraception Knowledge of contraceptive methods Knowledge of source of contraception Exposure to family planning messages in the media Exposure to family planning messages through personal contact Contact of non-users with family planning providers Discussion with husband/partner about family planning Husband/partners knowledge of wife's use of family planning Attitudes towards family planning Problems accessing care	Current marital status Children ever born and living Ever use of contraception Current use of contraception Unmet and met need for contraception	Wanted fertility rate Trends in contraceptive use Use of social marketing brands Source of contraception Cost of contraception Informed choice about contraception Problems with current method Discontinuation rate Reasons for discontinuation Intended future use Reasons for non-use Preferred method Knowledge of fertile period

The inclusion of unmarried adolescents varied. Three of the eleven countries (Indonesia, Timor-Leste and Vietnam) sampled 'ever-married women' only. The remaining countries included unmarried adolescents in the sampled population, however, most data were reported for 'all women', combining both married and unmarried women and not allowing for comparisons between these two groups. Some indicators were only reported for currently married women despite 'all women' being sampled. Five of the 40 indicators were disaggregated by both age and marital status to demonstrate outcomes for unmarried adolescents. However, only four countries (Marshall Islands, Samoa, Solomon Islands and Tuvalu) reported data for unmarried adolescents for one or more of these indicators.

### Adolescent sexual activity, fertility and unintended pregnancy

A significant proportion of women commence sexual activity during adolescence (Table 2). The median age of sexual debut for women aged 25-49 ranges from 17.3 to 21.9 years and is similar to age at first marriage. The proportion of women who commence sexual activity before the age of 15 varies considerably, from 0.5% in Tuvalu to 12.3% in Nauru. The proportion of adolescents who are currently sexually active (within the last four weeks) or have ever had sexual intercourse also varies considerably among countries that report data for 'all women'. Around half of adolescents in Marshall Islands, Nauru and Solomon Islands have ever had sex, and a considerable proportion commence sexual activity before the age of 15 years.

**Table 2:** Adolescent sexual activity - 'ever-married' and 'all women' (married and unmarried)

Country and survey year	Median age first marriage, females aged 25-49 (years)	Proportion females aged 15-19 currently married (%)	Median age sexual debut, females aged 25-49 (years)	Proportion females aged 25-49 sexual debut <15 years (%)	Proportion females aged 15-19 currently sexually active (%)	Proportion females aged 15-19 ever had sex (%)
***'Ever-married women'***						
Indonesia, 2007	19.8	12.8	19.7	1.3	*n/a*	*n/a*
Timor-Leste, 2003	21.4	11.2	20.7	-	*n/a*	*n/a*
Vietnam, 2002	21.1	4.1	-	-	*n/a*	*n/a*
						
***'All women'***						
Cambodia, 2005	20.1	9.8	20.4	2.0	8.3	10.8
Marshall Islands, 2007	19.6	1.2	17.3	11.6	29.3	64.5
Nauru, 2007	21.2	6.3	17.9	12.3	23.4	49.0
Papua New Guinea, 1996	19.9	18.2	-	-	-	-
Philippines, 2003	22.0	3.9	21.9	3.0	6.7	10.1
Samoa, 2009	23.6‡	2.7	-	-	-	-
Solomon Islands, 2007	19.9	9.7	18.2	12.1	21.2	51.9
Tuvalu, 2007	22.1	8.0	21.7†	0.5	9.1	14.2

†Includes women aged 20-49

‡Samoa reports 'Singulate Mean Age at Marriage'

**Figure 1:**
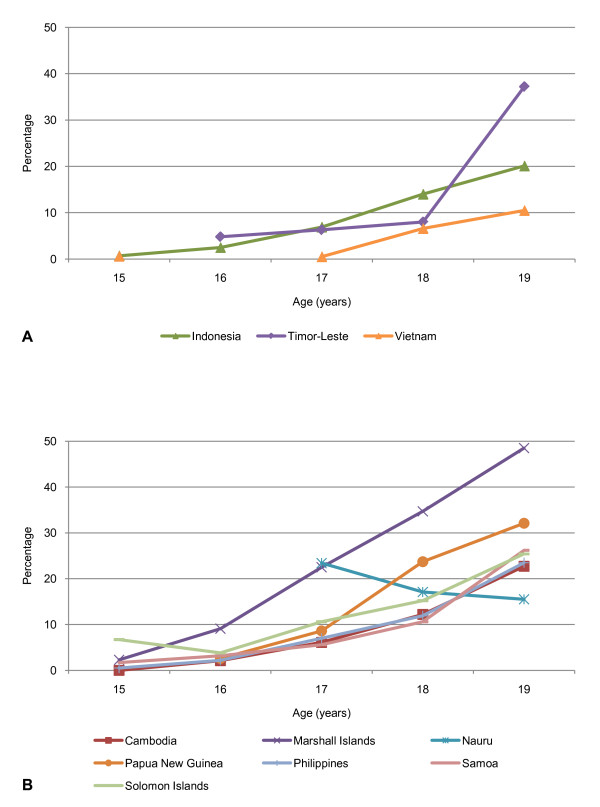
**Proportion of women aged 15-19 who have commenced childbearing at age *x *for A. 'ever-married women' and B. 'all women' (married and unmarried) (%)**.

The median age of first birth ranges from 20.2 to 23.4 years. Between 3.4 and 26.3% of women aged 15-19 are pregnant or have at least one previous birth (Table 3). By 19 years of age between 10.5 and 48.5% of adolescents have commenced childbearing (Figure 1). Adolescent fertility rates are high in most of the eleven countries, ranging from 25 to 138 births per 1000 women aged 15-19 years, and while they have declined in most countries compared with the 10 to 14 years preceding the survey, rates have remained relatively unchanged in Philippines, Samoa, Tuvalu and Vietnam, and have increased in Marshall Islands.

**Table 3:** Adolescent fertility - 'ever-married' and 'all women' (married and unmarried)

Country and survey year	Proportion females aged 15-19 who have commenced childbearing (%)	Median age first birth females aged 25-49 (years)	Adolescent fertility rate (births per 1000 women aged 15-19)	Adolescent fertility rate in the 10 to 14 years preceding the survey
***'Ever-married women'***				
Indonesia, 2007	8.5	21.5	51.0	67.0
Timor-Leste, 2003	14.5	23.0†	78.3	105.0‡
Vietnam, 2002	3.4	22.7	25.0	20.0*
***'All women'***				
Cambodia, 2005	7.8	22.0	47.0	85.0
Marshall Islands, 2007	26.3	20.2	138.0	105.0
Nauru, 2007	15.0	21.9	69.0	101.0
Papua New Guinea, 1996	13.8	21.0	77.0	104.0
Philippines, 2003	8.0	23.2	53.0	66.0
Samoa, 2009	9.4	23.4	44.0	50.0
Solomon Islands, 2007	11.9	21.1	67.0	95.0
Tuvalu, 2007	8.0	23.1	42.0	34.0

†Includes women aged 20-49

‡12-15 years preceding survey

*ASFR reported in the 1987 DHS

**Figure 2:**
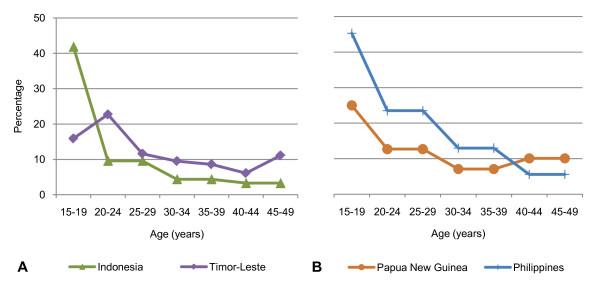
**Proportion of births to women aged 15-49 spaced less than 18 months for A. 'ever-married women' and B. 'all women' (married and unmarried) (%) **Cambodia, Marshall Islands, Nauru, Samoa, Solomon Islands, Tuvalu and Vietnam do not include data for adolescents for this indicator because of too few non-first births in the 15-19 age group.

A significant proportion of births to adolescents are spaced less than 18 months apart. For the four countries that include data on women aged 15-19 for this indicator, 15.9 to 45.3% of adolescent births were separated by less than 18 months, the highest proportion of all age groups except in Timor-Leste (Figure 2).

**Figure 3:**
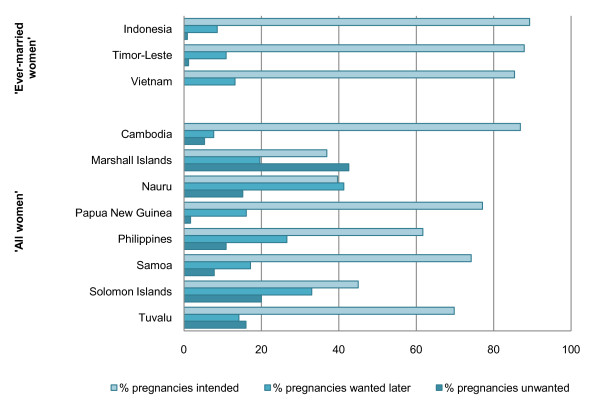
**Proportion of pregnancies intended, mistimed or unwanted for 'ever-married' and 'all women' (married and unmarried) aged 15-19 (%)**.

Between 36.9 and 89.3% of pregnancies to adolescents are intended, or 'wanted now' (Figure 3). A significant proportion of adolescent pregnancies are mistimed or unwanted. In Marshall Islands, Nauru and Solomon Islands more than half of women aged 15-19 report that their last pregnancy was unintended. These countries also report the highest proportion of adolescents who have ever had sex relative to the proportion who are currently married.

### Current use and unmet need for contraception

Current contraceptive prevalence, any method, among married adolescents varies greatly, ranging from 5.8 to 46.8%, with the lowest use reported in Timor-Leste (Table 4). The majority of adolescents currently using contraception are using a modern method. However, a relatively large proportion of adolescents in Cambodia, Nauru, Papua New Guinea, Philippines, Solomon Islands and Vietnam are relying on traditional (and presumably less effective) methods.

**Table 4:** Percent distribution of current contraceptive use by method for currently married and 'all women' (married and unmarried) aged 15-19 (%)

Country and survey year	Any method		Any modern method		Any traditional method	
	
	*Currently married*	*'All women'*	*Currently married*	*'All women'*	*Currently married*	*'All women'*
Cambodia, 2005	20.8	2.1	13.7	1.4	7.1	0.7
Indonesia, 2007	46.8	-	46.2	-	0.7	-
Marshall Islands, 2007	25.1	10.0	23.7	9.3	1.3	0.7
Nauru, 2007	*	8.5	*	3.8	*	4.7
Papua New Guinea, 1996	9.0	2.3	5.4	1.1	3.6	1.1
Philippines, 2003	25.6	2.4	13.2	1.3	12.3	1.1
Solomon Islands, 2007	19.5	7.2	12.8	5.2	6.7	2.1
Samoa, 2009	8.1	1.0	8.1	0.8	0.0	0.2
Timor-Leste, 2003	5.8	-	5.8	-	0.0	-
Tuvalu, 2007	*	2.7	*	2.7	*	0.0
Vietnam, 2002	22.8	-	14.1	-	8.6	-

* Figure not reported due to fewer than 25 cases

Current use of contraception is considerably lower among 'all women' (combining both married and unmarried adolescent women) compared with married women for those countries that report data for both groups. Marshall Islands, Nauru, Solomon Islands and Tuvalu also report contraceptive use for unmarried sexually active women, but only Marshall Islands and Solomon Islands report these data for adolescents: current use of any method for sexually active unmarried adolescents is 14.1% in Marshall Islands and 21.5% in Solomon Islands. Ninety and 65.6% of these women respectively are using a modern method.

**Figure 4:**
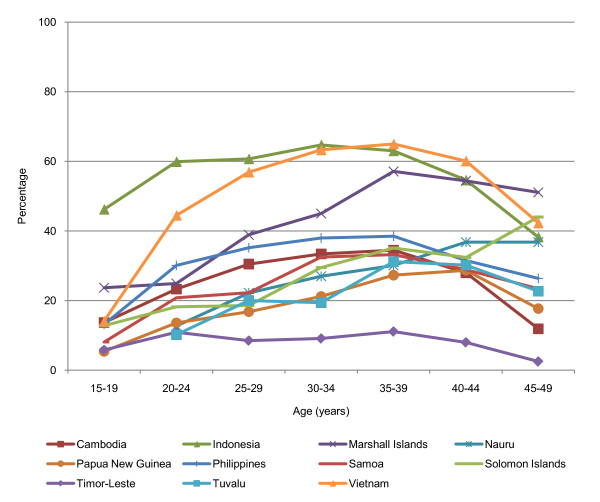
**Current contraceptive prevalence, modern method, currently married women aged 15-49 (%)**.

In all countries with data, the contraceptive prevalence (any, modern and traditional methods) is lowest for married adolescents compared with adults, except for women aged 45-49 (Figure 4). This age-related trend is also observable for data reported for 'all women' or unmarried sexually active women.

**Figure 5:**
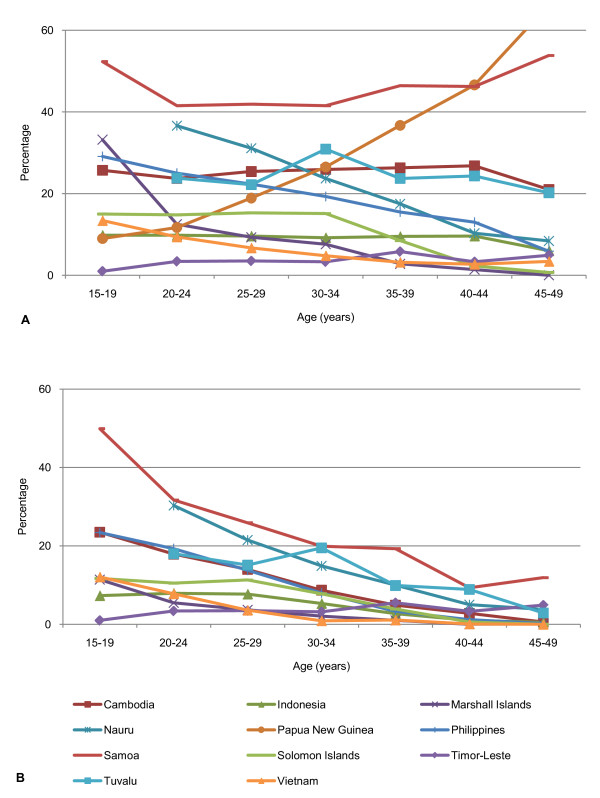
**Proportion of currently married women aged 15-49 with A. unmet need for contraception and B. unmet need for birth spacing (%) **Nauru and Tuvalu do not report data for women aged 15-19 due to fewer than 25 cases.

Low contraceptive prevalence may reflect a desire to become pregnant or an inability to access family planning, resulting in an unmet need for contraception. A woman is considered to have an unmet need if she is of reproductive age and able to become pregnant, is married or in a consensual union, wants to limit or delay pregnancy, and is not using a traditional or modern method of contraception. This includes women who are pregnant or have given birth in the last six months if the pregnancy was unintended [22]. Between 1.0 and 52.3% of married adolescents report an unmet need for contraception (Figure 5(a)). One to 49.9% of married adolescents also report an unmet need for birth spacing, that is they wish to delay their next pregnancy by two years or more but are not using any method of contraception (Figure 5(b)). Married women aged 15-19 have the highest unmet need of any age group in Indonesia, Marshall Islands, Philippines and Vietnam and among the highest unmet need in Cambodia, Samoa and Solomon Islands.

Some DHS also report source and cost of contraception, informed choice, problems with current method, reasons for and rate of discontinuation, intention for future use, reasons for non-use, and preferred methods. However, none of these indicators report data for women aged 15-19.

### Knowledge of family planning and access to information and services

**Figure 6:**
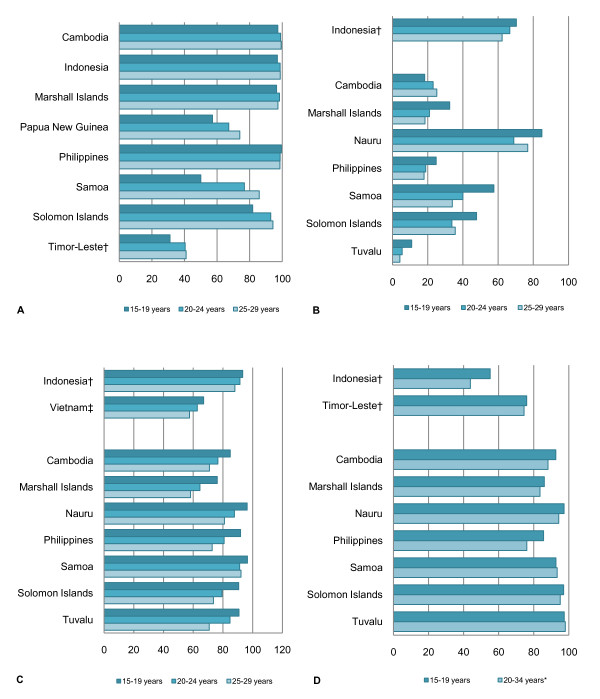
**Knowledge, exposure and access to information and services**. A. Proportion of currently married women reporting knowledge of at least one modern method of contraception (%). B. Proportion of women who have not seen or heard a family planning message in the media (%). C. Proportion of women not using contraception who did not discuss family planning with either a fieldworker or health service (%). D. Proportion of women who report at least one barrier to accessing health services (%) Data are for 'all women' (married and unmarried) unless otherwise indicated. †Includes 'ever-married women' only. ‡Includes currently married women only. * Philippines includes women aged 20-29.

The percentage of women aged 15-19 (currently or 'ever-married') with knowledge of a modern method of contraception ranges from 31.1 to 99.4% (Figure 6(a)). In countries with overall high knowledge of modern contraception, the difference between adolescents and older women appears to be minimal. However, in settings where overall knowledge is lower, a greater proportion of adolescents than older women cannot recognise or name a modern method. Papua New Guinea and Timor-Leste also report knowledge of a source of family planning: 48.8 and 21.4% of married adolescents respectively know a source of contraception, the lowest of all age groups except for women aged 45-49 in Timor-Leste.

Between 10.9 and 84.8% of adolescents report that they have not seen or heard a family planning message in the media (including radio, television, newspaper/magazine, poster or pamphlet) and the majority not using contraception have not discussed family planning with a health worker in the last 12 months (Figure 6(b) and 6(c)). In most countries, a larger proportion of adolescents than women aged 20-29 have not discussed or been exposed to family planning information. In addition, between 55.2 and 97.4% of adolescents report at least one barrier to accessing health services, a greater proportion than women aged 20-34 (Figure 6(d)). Problems accessing care include financial barriers, poor geographical access, lack of knowledge of services and concerns about availability of health workers and commodities. Problems related to needing permission, not wanting to go alone and concern that there would be no female provider are particularly noted by adolescents compared with older women.

## Discussion

DHS reports provide a large amount of useful information about adolescent fertility and family planning that is relevant to policymakers and programmers. All countries report data for adolescent fertility, contraceptive use and unmet need, and the majority also report data for adolescent sexual activity, knowledge and access to family planning information and services. However, DHS reports have been demonstrated to have some limitations [36]. These include the omission of young adolescents (10-14 years), the omission of unmarried adolescents or failure to report data for unmarried sexually active adolescents, and the failure to report age-disaggregated data for some important indicators, particularly contraceptive preferences and discontinuation. Data reporting broader sexual and reproductive health outcomes for adolescents, including sexually transmitted infections, HIV, and gender-based violence, are also required to inform comprehensive reproductive policy and programs. While beyond the scope of this review, the availability of these data in DHS reports has also been demonstrated to be limited [36].

### Unmarried adolescents

The inability to determine outcomes for unmarried sexually active adolescents from the reported data is significant, particularly as some countries report a high prevalence of currently or ever sexually active adolescents in the context of a low prevalence of adolescent marriage. In general, the countries that include data for 'all women' (married and unmarried) report different outcomes to those that only include 'ever-married women', suggesting that unmarried adolescents' health outcomes and access to information and services differ to married adolescent women. Other studies have indicated that unmarried adolescents experience unique barriers to accessing reproductive health services and have different outcomes in relation to contraceptive use, unintended pregnancy and abortion [3-7], therefore their inclusion in reports of national-level surveys is vital.

### Adolescent sexual activity, fertility and unintended pregnancy

A significant proportion of women commence sexual activity during adolescence. In the six countries that report data for 'all women' more than 10% of adolescents have ever had sex. In Marshall Islands, Nauru and Solomon Islands around half of adolescents have ever had sex, but less than 10% are married. Many adolescents, married and unmarried, are exposed to the risk of early pregnancy, highlighting the need for access to family planning information and services. Available data suggest this need is not being met, with a considerable proportion of adolescents commencing childbearing by the age of 19, a substantial number of adolescent births spaced less than 18 months apart, and a significant proportion of pregnancies unintended or unwanted.

A greater proportion of adolescent pregnancies are reportedly unwanted in countries that collect data for unmarried and married women compared with those that only include married women, particularly in those countries that report low adolescent marriage but high adolescent sexual activity. This could suggest that more unintended pregnancies occur among unmarried adolescents, although this was not able to be determined from the DHS reports due to the lack of data disaggregated by both age and marital status. An earlier review of adolescent childbearing in low income countries demonstrated that pregnancies to unmarried adolescents are much more likely to be unintended than married women [3], highlighting the need to report data for unmarried adolescents to inform policy and programs that aim to increase access to family planning.

Early pregnancy, intended or unintended, carries an increased risk of adverse health and socio-economic outcomes for women and their families and may result from poor access to information about family planning and the benefits of delaying first birth, poor access to reproductive health services, and socio-cultural expectations of early marriage and childbearing. Further research is required to better understand the factors that influence early childbearing and contraceptive use among married and unmarried adolescents in East Asia and the Pacific and identify potential targets for intervention.

### Contraceptive use and unmet need

The prevalence of modern methods of contraception among married adolescents is low in most countries. Low contraceptive use among young women is often considered to reflect a desire to become pregnant, particularly in settings where there is socio-cultural pressure to prove fertility [7,12,37]. However, the findings of this review suggest that a significant proportion of married adolescents want to delay or space their pregnancies but are unable to do so. This includes women who have already proven their fertility, but still report a high unmet need for contraception to space their next birth. The reasons for this are likely to be complex, and may include socio-cultural factors or limited choice of appropriate and acceptable methods for birth-spacing [5], and require further investigation.

There is very little data on contraceptive use and unmet need among unmarried sexually active women. Contraceptive prevalence is considerably lower when all adolescents (regardless of marital status) are included. This suggests that a large proportion of unmarried adolescents are not using contraception, although it can't be determined what proportion of these are sexually active from the DHS reports. Only Marshall Islands and Solomon Islands report data for this group, and contraceptive prevalence is low among unmarried sexually active adolescents in both countries. Use of any method is lower than for married adolescents in Marshall Islands, but higher in Solomon Islands. Other studies have demonstrated that unmarried adolescents have different patterns of contraceptive use compared to married women, often reporting higher contraceptive prevalence but also higher unmet need and higher rates of discontinuation [4,16,17,38]. Further research is warranted to explore the context-specific reasons for low contraceptive use and discontinuation among both married and unmarried adolescents, and to identify preferred contraceptive methods.

### Knowledge and access to information and services

Inadequate knowledge is one of the factors that contributes to low contraceptive use [4,39,40]. Married adolescents' knowledge of modern methods of contraception varies considerably between countries, and is limited in some settings. Countries that report the lowest levels of knowledge, Timor-Leste, Samoa, Solomon Islands and Papua New Guinea, also report the lowest contraceptive prevalence among married adolescents. As many women commence sexual activity during adolescence, reproductive health information needs to be provided from an early age, with evidence suggesting that this can have life-long protective health effects [39,41]. The proportion of adolescents who have been exposed to family planning messages in the media also varies substantially, and is low in some countries. Delivering family planning information through mass media, in addition to other promotion efforts including referral to services, has been associated with an increase in contraception uptake [42], so appropriate channels to better reach adolescents need to be explored.

The majority of adolescents not using contraception have not discussed family planning with a health worker and a considerable proportion report at least one barrier to accessing health care. Adolescents face unique barriers to accessing reproductive health information and services, contributing to low contraceptive use in this age group [12]. These include lack of decision-making power and access to or control over resources, socio-cultural norms regarding adolescent sexual behaviour and childbearing, and policy and legal restrictions [39,40,43,44]. The real and perceived skills, beliefs and attitudes of health workers can also affect the quality of information given to adolescents and their access to reproductive health services, including family planning [45,46]. Many of these factors are context-specific, highlighting the need for DHS data to be complemented by qualitative research. In addition, the information and service delivery preferences of adolescents require further investigation to identify how these barriers may be overcome.

### Adolescents compared with adult women

In all eleven countries adolescents are less protected against unintended pregnancy than older women, with contraceptive use considerably lower in this age group than all others, except women over the age of 44. This is consistent with previous studies of contraceptive use in other low income countries [5,6]. In most countries adolescents also have higher unmet need for contraception, less knowledge, and poorer access to information and services than older women. These findings suggest that efforts to scale up reproductive health interventions, including increasing the uptake of family planning, do not necessarily reach adolescents and that targeted responses are required. While there is a growing body of evidence regarding youth-targeted programs to deliver reproductive health information and services [12,47-49] there is a need to support more rigorous evaluations to identify effective approaches.

### Limitations

This review has a number of limitations. Some surveys were conducted more than five to ten years ago and it is likely that adolescent fertility and contraceptive use have changed in that time. Only data available in DHS reports were included to reflect data readily accessible to policymakers and programmers. Analysis of primary data was beyond the scope of this review, however, further analysis is likely to yield useful information. This would be particularly relevant to those indicators not disaggregated by age, such as contraceptive preferences and discontinuation, as well as exploring outcomes for unmarried sexually active adolescents. In addition, sampling populations, survey questions and reporting of age-disaggregated data and marital status vary between countries for some indicators, limiting the ability to make country comparisons.

## Conclusion

DHS reports provide much useful data accessible to policymakers and programmers; however they are limited by the failure to report data for unmarried sexually active adolescents or report age-disaggregated data for some indicators. Available data indicate that adolescent sexual activity and pregnancy are common in East Asia and the Pacific in the context of low contraceptive prevalence. Adolescents also appear to have lower use and higher unmet need for contraception, poorer knowledge of family planning and less access to information and services than older women.

The prevention of adolescent pregnancy is an integral component of efforts to improve maternal health and ensure universal access to reproductive health, but it cannot be assumed that adolescents will automatically benefit from policies and programs that are aimed at the general population. Further research is required to better understand the barriers that both married and unmarried adolescents face accessing reproductive health information and services, and their information and service delivery preferences, so that interventions can be effectively targeted to meet their needs. In addition, greater investment is needed to support rigorous evaluation of strategies that target adolescents so that effective approaches can be identified.

## Competing interests

The authors declare that they have no competing interests.

## Authors' contributions

NG and MC designed the initial review. EK, PA and NG reviewed the DHS reports and conducted the data entry and analysis. EK prepared the first draft of the paper. All authors made substantial comments and contributions to subsequent drafts and approved the final version submitted for publication.
